# Perceptions of Institutional Engagement and Inclusion by Sexual Orientation and Gender Identity

**DOI:** 10.1001/jamanetworkopen.2025.13772

**Published:** 2025-06-04

**Authors:** Stefanie N. Hinkle, Chelsea C. Okeh, Ernesto Ulloa-Pérez, Ashika Mani, Eve J. Higginbotham, Rosemary Thomas, Matthew D. Kearney, Corrinne Fahl, Enrique F. Schisterman, Shefali S. Verma, Roy Hamilton, Sunni L. Mumford

**Affiliations:** 1 Department of Biostatistics, Epidemiology and Informatics, Perelman School of Medicine, University of Pennsylvania, Philadelphia; 2 Department of Obstetrics and Gynecology, Perelman School of Medicine, University of Pennsylvania, Philadelphia; 3 Department of Pathology and Laboratory Medicine, Perelman School of Medicine, University of Pennsylvania, Philadelphia; 4 Office for Inclusion, Diversity, and Equity, Perelman School of Medicine, University of Pennsylvania, Philadelphia (former affiliation); 5 Leonard Davis Institute for Health Economics, University of Pennsylvania, Philadelphia; 6 Department of Ophthalmology, Perelman School of Medicine, University of Pennsylvania, Philadelphia; 7 Penn Medicine Center for Health Equity Advancement and Program for LGBTQ+ Health, University of Pennsylvania Health System, Philadelphia; 8 Department of Family Medicine and Community Health, Perelman School of Medicine, University of Pennsylvania, Philadelphia; 9 Office of the Dean, Perelman School of Medicine, University of Pennsylvania, Philadelphia; 10 Department of Neurology, Perelman School of Medicine, University of Pennsylvania, Philadelphia

## Abstract

**Importance:**

There is a paucity of research regarding the experiences of self-identified lesbian, gay, bisexual, and transgender (LGBT+) individuals in academic medicine.

**Objective:**

To examine LGBT+ individuals’ perceptions of institutional engagement and workplace inclusivity.

**Design, Setting, and Participants:**

In this survey study, in 2015, 2018, 2021, and 2023, the Diversity Engagement Survey (DES) supplemented with specific questions about LGBT+ visibility and engagement in the workplace was sent to faculty, students and trainees, and staff at a single academic medical center in Pennsylvania.

**Exposures:**

Sexual orientation was self-identified as heterosexual; lesbian, gay, or bisexual [LGB]; or other. Gender identity was self-identified as men; transgender, queer, or nonbinary [TQNB]; women; or other or unknown.

**Main Outcomes and Measures:**

Workplace culture inclusivity was assessed using 8 validated DES constructs summarized into 3 scores: perceptions of shared vision and purpose, camaraderie, and appreciation of contributions to the institution. Three questions evaluated statements about institutional welcoming of LGBT+ individuals, comfort working with LGBT+ colleagues, and LGBT+ institutional visibility. Potential attrition was assessed through a question about job change considerations due to inappropriate, disruptive, or unprofessional behavior by a coworker or supervisor. Results were weighted to account for nonresponse.

**Results:**

Among 23 708 respondents (15.9% of approximately 149 500 survey recipients), 2068 (8.7%) identified as LGB and 169 (0.7%) as TQNB. Compared with heterosexual respondents, LGB respondents reported lower institutional engagement (vision and purpose: adjusted difference (AD), −1.2 [95% CI, −1.6 to −0.9]; camaraderie: AD, −1.1 [95% CI, −1.3 to −0.9]; appreciation: AD, −0.9 [95% CI, −1.1 to −0.6]) and were less likely to agree with statements of LGBT+ institutional inclusivity (welcoming: adjusted relative ratio [ARR], 0.88 [95% CI, 0.85-0.90]; visibility: ARR, 0.90 [95% CI, 0.86-0.94]). Compared with men, TQNB respondents reported lower engagement (vision and purpose: AD, −4.1 [95% CI, −5.5 to −2.6]; camaraderie: AD, −3.2 [95% CI, −4.1 to −2.3]; appreciation: AD, −2.6 [95% CI, −3.5 to −1.7]) and were less likely to agree with LGBT+ institutional inclusivity statements (welcoming: ARR, 0.65 [95% CI, 0.53-0.80]; visibility: ARR, 0.78 [95% CI, 0.61-1.00]). LGB (vs heterosexual; ARR, 1.26 [95% CI, 1.15-1.38]) and TQNB (vs men; ARR, 1.48 [95% CI, 1.17-1.88]) respondents were more likely to report job change consideration.

**Conclusions and Relevance:**

In this survey study, the findings demonstrated a need for focused and subgroup-specific intentional initiatives to optimize productivity and improve workplace culture, sense of belonging, and retention for self-identified sexual and gender minority individuals within academic medical communities.

## Key Points

**Question:**

Does institutional belonging and engagement vary by sexual orientation and gender identity in an academic medical community?

**Findings:**

In this survey study of 23 708 respondents, self-identified sexual or gender minority individuals reported significantly lower institutional engagement; were significantly less likely to agree there was institutional visibility or welcoming of lesbian, gay, bisexual, and transgender individuals; and were more likely to consider changing jobs compared with non–sexual or gender minority counterparts.

**Meaning:**

The findings suggest that greater specificity and intentionality are needed for effective initiatives to improve the environment and engagement for sexual or gender minority individuals in academic medical communities.

## Teaser

This survey study in an academic medical center assesses differences in perception of workplace engagement and inclusivity of lesbian, gay, bisexual, and transgender individuals by respondents’ self-identified sexual orientation and gender.

## Introduction

Increasing initiatives focused on inclusion, diversity, and equity has been a priority over the past decade in academic medical communities, especially as data have demonstrated improvements in patient care and satisfaction with a diverse workforce.^1,2,3,4^ Between 2019 and 2022, approximately 4% of medical faculty identified as lesbian, gay, or bisexual (LGB).^5^ However, the impact of inclusion, diversity, and equity initiatives on sexual and gender minority individuals is largely unknown. Despite recent external pressures to abandon efforts to strive for inclusive excellence, the need remains to reinforce efforts to optimize productivity and inclusive engagement of all individuals in the workforce.

The Diversity Engagement Survey (DES) was designed to measure employee engagement within academic medicine.^6^ In 2015, the DES was validated, and Person et al^6^ found that sexual and gender minority respondents, compared with heterosexual respondents, had lower levels of engagement. Additionally, in 2022, limited data from the Association of American Medical Colleges suggested that LGB faculty were less likely to report feeling respected in the workplace compared with heterosexual colleagues.^5^ This disparity in workplace respect may contribute to broader mental health challenges, as burnout, depression, and anxiety are common challenges for physicians who identify as sexual or gender minority individuals.^7^ Burnout, harassment, and a lack of collegiality and peer connections contribute to physician turnover.^8,9^

In 2012, the University of Pennsylvania (Penn) Perelman School of Medicine developed the interprofessional Penn Medicine Program for Lesbian, Gay, Bisexual, and Transgender (LGBT) Health to improve the care of LGBT+ populations.^10^ The program includes 5 focus areas, including institutional climate and visibility, health education, research, patient care, and community outreach, each having attained specific achievements since the start of the program.^10^ More details on the timeline of initiatives at Penn are in the eFigure in Supplement 1. One achievement of the institutional climate and visibility focus area was to add custom LGBT+ visibility questions and other questions monitoring the culture at Penn to Penn’s DES.

Our objectives were to assess (1) workplace engagement across sexual orientation and gender identity, (2) inclusivity for sexual and gender minority community members and how this perception varies according to respondent gender identity and sexual orientation, and (3) whether the overall perception of institutional culture and likelihood of considering leaving the job due to harassment differs by gender identity or sexual orientation. As a secondary objective, we examined whether these measures varied across the study period from 2015 to 2023. This work built on prior work in the field by distinctly examining sexual orientation and gender identity as separate demographics as well as the intersectionality of sexual orientation and gender identity to provide an in-depth focus on engagement of sexual and gender minority members of the community and understand how the community as a whole views inclusivity, comfort, and visibility of LGBT+ members.

## Methods

In this survey study, we analyzed data collected from the Penn DES in 2015, 2018, 2021, and 2023.^6^ Specifically, DES questionnaires were sent to faculty, staff, trainees, and students at 3 health systems, 2 health professional and graduate schools (Perelman School of Medicine, School of Nursing), and affiliated clinical facilities within Penn. The DES collected demographic information, job position, and questions related to institutional engagement and inclusion. All participants provided informed consent by voluntarily completing a confidential survey, in accordance with institutional review board–approved procedures, with assurances that responses were deidentified, reported in aggregate, and stored securely for potential future research. Survey completion was voluntary, and no incentives were offered. The Penn institutional review board approved the protocol for each year of the survey and determined that the study was exempt from review as it used only deidentified data. The study adhered to the American Association for Public Opinion Research (AAPOR) Best Practices for Survey Research reporting guideline.

### Outcome Measures

Institutional engagement was assessed using the standard 22 DES questions scored on a 5-point Likert scale (1 indicating strongly disagree and 5, strongly agree), generating scores for 8 factors: common purpose, trust, appreciation of individual attributes, sense of belonging, access to opportunity, equitable reward and recognition, cultural competence, and respect. These factors were grouped into 3 validated constructs (eTable 1 in Supplement 1):^6,11^ vision and purpose (common purpose, access to opportunity, equitable reward and recognition, and cultural competence), camaraderie (trust, sense of belonging), and appreciation (appreciation of individual attributes, respect).

Five additional survey items custom to the Penn survey were used. First, we assessed institutional inclusivity for sexual and gender minority individuals: (1) “My institution is welcoming of lesbian, gay, bisexual, and transgender individuals”; (2) “I am comfortable working with my lesbian, gay, bisexual, and transgender colleagues”; and (3) “There is lesbian, gay, bisexual, and transgender visibility at my institution.” Of note, the questions specifically focused on lesbian, gay, bisexual, and transgender individuals, and herein, *LGBT+* is used, with the addition of the “+” to include any other related identities that were not captured by this wording. We acknowledge that the acronym is continuously evolving and other acronyms may be more inclusive, but we used *LGBT+* to follow the wording of our survey. We use *LGB* when specifically focusing on sexual orientation. We also examined general institutional culture with the following item: “The culture of my institution values respect and tolerance for all.” Last, we examined job change considerations due to harassment with the following item: “I have considered changing jobs due to inappropriate, disruptive, or unprofessional behavior by a coworker or supervisor.” For brevity, we refer to these 5 items as the welcoming, comfort, visibility, culture, and job change items, respectively.

All questions were scored on a 5-point Likert-type scale, with responses dichotomized as “strongly agree” or “agree” vs all other options. If respondents chose “unable to evaluate,” the response was treated as missing.

### Self-Identified Sexual Orientation and Gender Identity

Respondents’ self-identified sexual orientation and gender identity were assessed through 2 questions, with small variations over survey years (eTable 2 in Supplement 1). Sexual orientation was assessed as heterosexual, LGB, or other or unknown. Gender identity was assessed as men; transgender, queer, or nonbinary (TQNB); women; or other or unknown. Those who did not respond were categorized as having unknown gender identity. We also assessed the intersectionality of sexual orientation and gender identity.

### Covariates

Covariates incorporated across all analyses included generational age group, self-identified race and ethnicity, location of main affiliation, position at main affiliation, and year. Race and ethnicity were included in the analysis as social determinants of health, recognizing their role in exposure to structural inequities; categories were Asian, Hispanic or Latino, non-Hispanic Black, non-Hispanic White, multiracial or multiethnic, and other or unknown (American Indian or Alaska Native, Native Hawaiian or Other Pacific Islander, and other written-in responses).

### Weighting for Nonresponse

To account for potential selection bias due to nonresponse,^12^ we weighted survey responses so that the overall frequencies of main affiliation, gender, and race and ethnicity were as reflective as possible of the overall Perelman School of Medicine population in each survey year, based on data from the Penn Community Survey (additional details are in the eMethods in Supplement 1). The weights were obtained using iteratively proportional fitting.^13^

### Statistical Analysis

Demographic and work-related characteristics were compared by survey year using descriptive statistics. Primary regression analyses focused on estimating differences across sexual orientation, gender identity, and the intersectionality of sexual orientation and gender identity. We analyzed the 3 continuous engagement constructs (ie, vision and purpose, camaraderie, and appreciation) using weighted linear regression. We analyzed 5 binary items (ie, welcoming, comfort, visibility, culture, and job change) using weighted Poisson regression with robust SEs. We estimated unadjusted models and models adjusted for year, generational age group (birth year of 1922-1944, 1945-1964, 1965-1980, 1981 or later, or unknown), race and ethnicity, main affiliation, and primary work location. All analyses accounted for nonresponse as described. Missing data were analyzed as a separate group (eg, age, gender) or combined with the “other” category. Students were not included in the job change analysis.

We conducted 2 secondary analyses. First, we stratified associations to assess differences by job position. Second, we evaluated whether associations changed across survey years. Due to a limited number of TQNB respondents, we only assessed stratified analyses by sexual orientation. We tested for changes between 2015 and 2023 in adjusted differences between each group and the reference. All analyses were conducted using RStudio, version 4.1.2 (RStudio, PBC).

## Results

### Sample Characteristics

Of the approximately 149 500 individuals to whom the survey was sent across all survey years, a total of 23 708 completed the survey (15.9%). Overall, 19 287 respondents (81.4%) identified as heterosexual, 2068 (8.7%) as LGB, and 2353 (9.9%) as other sexual orientation or did not respond (Table 1). The proportion of respondents who identified as LGB in 2015 was 288 of 3042 (9.5%) and in 2023 was 518 of 5225 (9.9%). Overall, 5977 participants (25.2%) identified as men, 169 (0.7%) as TQNB, 16 914 (71.3%) as women, and 648 (2.7%) as other gender or did not respond. The proportion of respondents identifying as TQNB was 14 of 3042 (0.5%) in 2015 and 66 of 5225 (1.3%) in 2023. Respondents’ generational age groups were roughly uniform, with 5922 (25.0%) born from 1945 to 1964, 8390 (35.4%) from 1965 to 1980, and 8528 (36.0%) in 1981 or later; few had a birth year between 1922 and 1944 (150 [0.6%]). A total of 1851 respondents (7.8%) identified as Asian, 3677 (15.5%) as Black, 918 (3.9%) as Hispanic or Latino, 14 912 (62.9%) as White, and 857 (3.6%) as multiracial or multiethnic; 1493 (6.3%) reported other or unknown race and ethnicity. Weighted distributions of respondent characteristics to address nonresponse are shown in eTable 3 in Supplement 1. After weighting, 80% of respondents identified as heterosexual, 10% as LGB, and 10% as another sexual orientation or did not respond; 56% of respondents identified as women, 40% as men, 1% as TQNB, and 3% as another gender or did not respond.

**Table 1.** Demographic and Work-Related Characteristics of Survey Respondents

Characteristic	Respondents, No. (%)				
	Overall (N = 23 708)	2015 (n = 3042)	2018 (n = 5022)	2021 (n = 10 419)	2023 (n = 5225)
Sexual orientation					
Heterosexual	19 287 (81.4)	2647 (87.0)	4184 (83.3)	8277 (79.4)	4179 (80.0)
LGB	2068 (8.7)	288 (9.5)	428 (8.5)	834 (8.0)	518 (9.9)
Other or unknown^a^	2353 (9.9)	107 (3.5)	410 (8.2)	1308 (12.6)	528 (10.1)
Gender identity					
Men	5977 (25.2)	1080 (35.5)	1194 (23.8)	2372 (22.8)	1331 (25.5)
TQNB	169 (0.7)	14 (0.5)	30 (0.6)	59 (0.6)	66 (1.3)
Women	16 914 (71.3)	1914 (62.9)	3688 (73.4)	7642 (73.3)	3670 (70.2)
Other or unknown^a^	648 (2.7)	34 (1.1)	110 (2.2)	346 (3.3)	158 (3.0)
Intersectionality					
Heterosexual					
Men	4853 (20.5)	920 (30.2)	955 (19.0)	1910 (18.3)	1068 (20.4)
TQNB	33 (0.1)	6 (0.2)	6 (0.1)	14 (0.1)	7 (0.1)
Women	14 229 (60.0)	1711 (56.2)	3182 (63.4)	6263 (60.1)	3073 (58.8)
LGB					
Men	742 (3.1)	130 (4.3)	156 (3.1)	280 (2.7)	176 (3.4)
TQNB	120 (0.5)	8 (0.3)	22 (0.4)	37 (0.4)	53 (1.0)
Women	1175 (5.0)	150 (4.9)	244 (4.9)	499 (4.8)	282 (5.4)
Other or unknown^a^	2556 (10.8)	117 (3.8)	457 (9.1)	1416 (13.6)	566 (10.8)
Birth year					
1922-1944	150 (0.6)	62 (2.0)	27 (0.5)	48 (0.5)	13 (0.2)
1945-1964	5922 (25.0)	784 (25.8)	1443 (28.7)	2654 (25.5)	1041 (19.9)
1965-1980	8390 (35.4)	913 (30.0)	1806 (36.0)	3730 (35.8)	1941 (37.1)
1981 or later	8528 (36.0)	1234 (40.6)	1629 (32.4)	3610 (34.6)	2055 (39.3)
Unknown^a^	718 (3.0)	49 (1.6)	117 (2.3)	377 (3.6)	175 (3.3)
Race and ethnicity					
Asian	1851 (7.8)	355 (11.7)	355 (7.1)	740 (7.1)	401 (7.7)
Hispanic or Latino	918 (3.9)	125 (4.1)	169 (3.4)	423 (4.1)	201 (3.8)
Non-Hispanic Black	3677 (15.5)	332 (10.9)	719 (14.3)	1809 (17.4)	817 (15.6)
Non-Hispanic White	14 912 (62.9)	1952 (64.2)	3274 (65.2)	6488 (62.3)	3198 (61.2)
Multiracial or multiethnic	857 (3.6)	152 (5.0)	195 (3.9)	300 (2.9)	210 (4.0)
Other or unknown^a^^,^^b^	1493 (6.3)	126 (4.1)	310 (6.2)	659 (6.3)	398 (7.6)
Main affiliation position					
Staff	13 495 (56.9)	1226 (40.3)	2798 (55.7)	6441 (61.8)	3030 (58.0)
Standing or associated faculty^c^	4735 (20.0)	856 (28.1)	1177 (23.4)	1510 (14.5)	1192 (22.8)
Student or fellow^d^	1735 (7.3)	836 (27.5)	277 (5.5)	361 (3.5)	261 (5.0)
Other or unknown^a^	3743 (15.8)	124 (4.1)	770 (15.3)	2107 (20.2)	742 (14.2)
Location of main affiliation					
Pennsylvania Hospital	1768 (7.5)	45 (1.5)	396 (7.9)	890 (8.5)	437 (8.4)
Perelman School of Medicine	2510 (10.6)	1236 (40.6)	389 (7.7)	531 (5.1)	354 (6.8)
Philadelphia VA Medical Center	60 (0.3)	25 (0.8)	7 (0.1)	18 (0.2)	10 (0.2)
School of Nursing	117 (0.5)	117 (3.8)	0	0	0
University of Pennsylvania–affiliated hospital	12 864 (54.3)	1304 (42.9)	2560 (51.0)	5857 (56.2)	3143 (60.2)
Other or unknown^a^	6389 (26.9)	315 (10.4)	1670 (33.3)	3123 (30.0)	1281 (24.5)

Abbreviations: LGB, lesbian, gay, or bisexual; TQNB, transgender, queer, or nonbinary; VA, Veterans Affairs.

^a^ Unknown includes respondents who did not select a choice for the respective question.

^b^ Other race and ethnicity includes American Indian or Alaska Native, Native Hawaiian or Other Pacific Islander, and other written-in responses.

^c^ Includes leadership.

^d^ Includes undergraduate students, graduate students, professional students, interns, residents, clinical fellows, and postdoctoral fellows.

### Perceptions of Institutional Engagement

**Figure 1.**
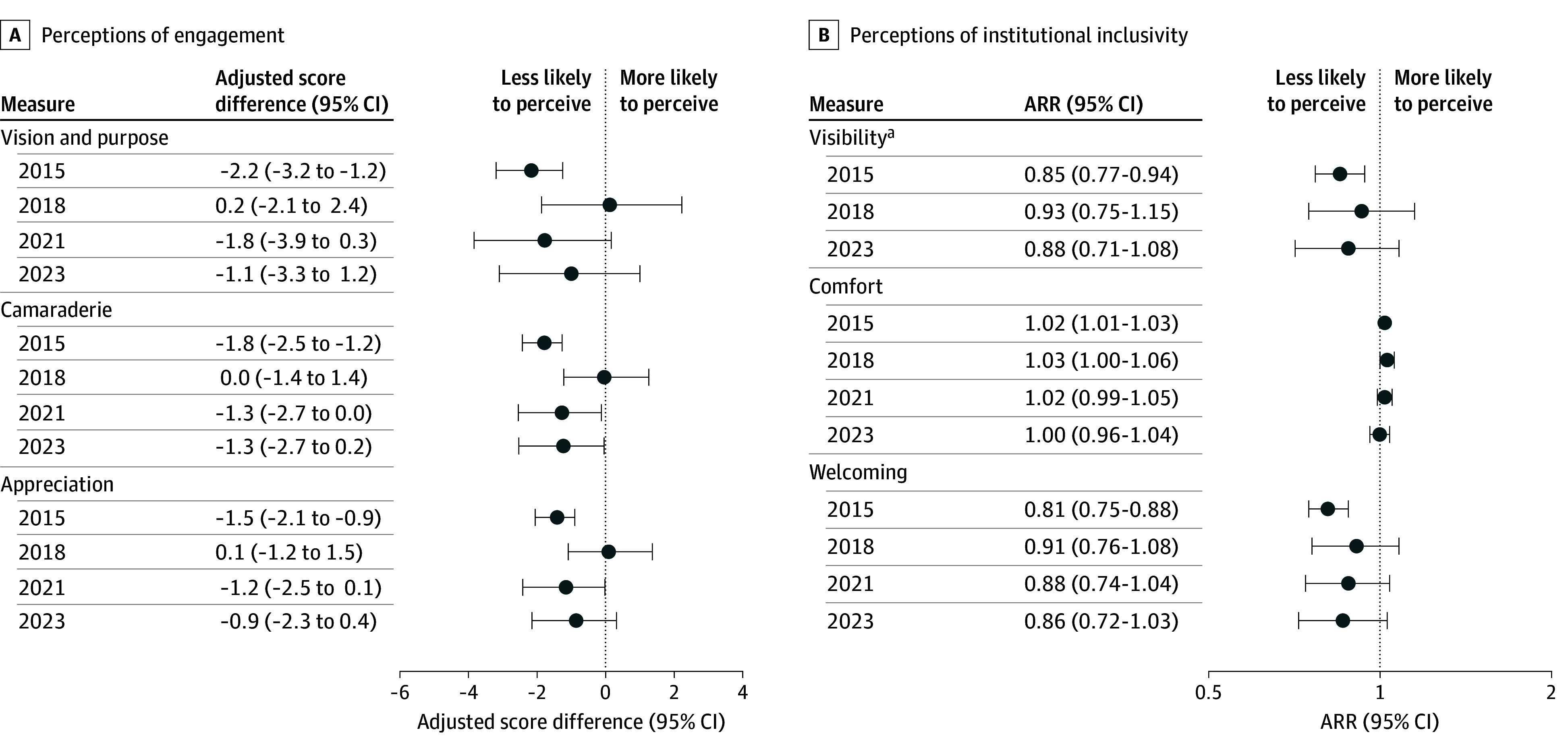
Differences in Perceptions of Engagement and Lesbian, Gay, Bisexual, and Transgender (LGBT+) Institutional Inclusivity by Sexual Orientation Across All Survey Years Data for respondents who reported “other” sexual orientation or were missing sexual orientation information are presented in eTables 5 and 7 in Supplement 1. Models were adjusted for survey year, generational age group, race and ethnicity, main affiliation, primary work location, and gender identity. All models were weighted by race and ethnicity, job position, and sex to account for nonresponse. A, Survey items were scored on a 5-point Likert scale, with higher scores indicating greater likelihood of perceiving the measure. ARR indicates adjusted relative ratio. ^a^The visibility question was not asked in 2021.

On average, respondents identifying as LGB had lower adjusted scores across vision and purpose (difference, −1.2 points; 95% CI, −1.6 to −0.9 points), camaraderie (difference, −1.1 points; 95% CI, −1.3 to −0.9 points), and appreciation (difference, −0.9 points; 95% CI, −1.1 to −0.6 points) compared with respondents identifying as heterosexual (Table 2). Respondents identifying as women compared with men had lower adjusted scores for vision and purpose (difference, −1.3 points; 95% CI, −1.6 to −1.0 points), camaraderie (difference, −0.8 points; 95% CI, −1.0 to −0.6 points), and appreciation (difference, −0.7 points; 95% CI, −0.9 to −0.6 points), while respondents identifying as TQNB compared with men had substantially lower scores for vision and purpose (difference, −4.1 points; 95% CI, −5.5 to −2.6 points), camaraderie (difference, −3.2 points; 95% CI, −4.1 to −2.3 points), and appreciation (difference, −2.6 points; 95% CI, −3.5 to −1.7 points). Respondents identifying as both TQNB and LGB had the lowest adjusted scores, with differences of −6.1 points (95% CI, −7.7 to −4.4 points) for vision and purpose, −4.6 points (95% CI, −5.6 to −3.5 points) for camaraderie, and −3.9 points (95% CI, −4.7 to −3.0 points) for appreciation compared with respondents identifying as heterosexual men. Differences were largest among staff followed by students and then faculty (eTable 4 in Supplement 1). No meaningful changes were observed in any measures from 2015 to 2023 (Figure 1A and eTable 5 in Supplement 1).

**Table 2.** Associations of Respondent Sexual Orientation and Gender Identity With Measures of Institutional Engagement

Characteristic	Vision and purpose		Camaraderie		Appreciation	
	Mean (SE) score^a^	Adjusted difference (95% CI)^b^	Mean (SE) score^a^	Adjusted difference (95% CI)^b^	Mean (SE) score^a^	Adjusted difference (95% CI)^b^
Sexual orientation						
Heterosexual	39.4 (7.5)	[Reference]	23.6 (4.8)	[Reference]	24.0 (4.7)	[Reference]
LGB	38.1 (7.8)	−1.2 (−1.6 to −0.9)	22.3 (5.1)	−1.1 (−1.3 to −0.9)	23.0 (4.9)	−0.9 (−1.1 to −0.6)
Other or unknown	37.1 (8.7)	−0.5 (−0.9 to −0.1)	22.0 (5.5)	−0.4 (−0.6 to −0.1)	22.4 (5.6)	−0.5 (−0.7 to −0.3)
Gender identity						
Men	40.0 (7.7)	[Reference]	24.0 (4.9)	[Reference]	24.4 (4.8)	[Reference]
TQNB	35.1 (8.6)	−4.1 (−5.5 to −2.6)	19.8 (5.8)	−3.2 (−4.1 to −2.3)	21.1 (5.5)	−2.6 (−3.5 to −1.7)
Women	38.9 (7.6)	−1.3 (−1.6 to −1.0)	23.2 (4.9)	−0.8 (−1.0 to −0.6)	23.7 (4.8)	−0.7 (−0.9 to −0.6)
Other or unknown	35.8 (8.4)	−0.8 (−1.8 to 0.1)	20.8 (5.6)	−0.8 (−1.4 to −0.3)	21.1 (5.6)	−1.0 (−1.6 to −0.4)
Intersectionality						
Heterosexual						
Men	40.4 (7.5)	[Reference]	24.3 (4.7)	[Reference]	24.6 (4.7)	[Reference]
TQNB	39.3 (8.3)	−0.7 (−3.8 to 2.5)	22.8 (4.9)	−1.0 (−2.7 to 0.7)	23.9 (4.7)	−0.4 (−2.1 to 1.2)
Women	39.1 (7.5)	−1.4 (−1.7 to −1.1)	23.4 (4.8)	−0.9 (−1.1 to −0.7)	23.8 (4.8)	−0.8 (−0.9 to −0.7)
LGB						
Men	39.0 (8.0)	−1.4 (−2.2 to −0.6)	23.0 (5.1)	−1.1 (−1.6 to −0.6)	23.7 (4.9)	−0.9 (−1.2 to −0.6)
TQNB	34.0 (8.5)	−6.1 (−7.7 to −4.4)	19.1 (5.7)	−4.6 (−5.6 to −3.5)	20.5 (5.6)	−3.9 (−4.7 to −3.0)
Women	37.9 (7.4)	−2.4 (−3.1 to −1.7)	22.2 (4.9)	−1.9 (−2.3 to −1.4)	22.9 (4.7)	−1.6 (−1.9 to −1.2)
Other or unknown	37.2 (8.6)	−1.5 (−2.1 to −0.9)	22.0 (5.5)	−1.0 (−1.4 to −0.7)	22.4 (5.6)	−1.1 (−1.4 to −0.9)

Abbreviations: LGB, lesbian, gay, or bisexual; TQNB, transgender, queer, or nonbinary.

^a^ Higher scores indicate greater agreement with the measure.

^b^ Models were adjusted for survey year, generational age group, race and ethnicity, main affiliation, job position, and primary work location. Gender identity models were additionally adjusted for sexual orientation, and sexual orientation models were additionally adjusted for gender identity. All models were weighted by race and ethnicity, job position, and sex to account for nonresponse.

### Perceptions of LGBT+ Institutional Inclusivity

Respondents identifying as LGB were less likely to agree their institution was welcoming of LGBT+ individuals (adjusted relative ratio [ARR], 0.88; 95% CI, 0.85-0.90) and to agree there was visibility of LGBT+ individuals (ARR, 0.90; 95% CI, 0.86-0.94) compared with heterosexual respondents (Table 3). They were more likely than heterosexual respondents to report comfort working with LGBT+ colleagues (ARR, 1.02; 95% CI, 1.01-1.03). Compared with men, TQNB respondents were less likely to agree their institution was welcoming of LGBT+ individuals (ARR, 0.65; 95% CI, 0.53-0.80) and to agree there was visibility of LGBT+ individuals (ARR, 0.78; 95% CI, 0.61-1.00). Respondents who were both LGB and TQNB were less likely to feel their institution was welcoming of LGBT+ individuals (ARR, 0.56; 95% CI, 0.47-0.67) and to perceive visibility of LGBT+ individuals (ARR, 0.66; 95% CI, 0.54-0.80) compared with heterosexual men. Associations of perceptions differed most among staff followed by students (eTable 6 in Supplement 1). No meaningful changes were observed from 2015 to 2023 (Figure 1B and eTable 7 in Supplement 1).

**Table 3.** Associations of Respondent Sexual Orientation and Gender Identity With Measures of LGBT+ Institutional Inclusivity and Institutional Culture

Characteristic	Welcoming^a^		Comfort^b^		Visibility^c^		Culture^d^	
	Respondents, No. (%)^e^	ARR (95% CI)^f^	Respondents, No. (%)^e^	ARR (95% CI)^f^	Respondents, No. (%)^e^	ARR (95% CI)^f^	Respondents, No. (%)^e^	ARR (95% CI)^f^
Sexual orientation								
Heterosexual	15 898 (83.9)	1 [Reference]	17 848 (94.2)	1 [Reference]	7895 (41.7)	1 [Reference]	12 091 (63.8)	1 [Reference]
LGB	1860 (75.9)	0.88 (0.85-0.90)	2379 (97.1)	1.02 (1.01-1.03)	1017 (41.5)	0.90 (0.86-0.94)	1431 (58.4)	0.93 (0.90-0.96)
Other or unknown	1629 (70.5)	0.93 (0.90-0.96)	1939 (83.9)	0.96 (0.94-0.98)	672 (29.1)	0.93 (0.88-0.98)	1187 (51.4)	0.95 (0.91-0.99)
Gender identity								
Men	7804 (82.1)	1 [Reference]	8900 (93.6)	1 [Reference]	4024 (42.3)	1 [Reference]	6159 (64.8)	1 [Reference]
TQNB	88 (52.1)	0.65 (0.53-0.80)	161 (95.3)	0.99 (0.84-1.16)	64 (37.9)	0.78 (0.61-1.00)	61 (36.1)	0.75 (0.58-0.97)
Women	11 070 (82.7)	0.99 (0.96-1.02)	12 591 (94.1)	1.01 (0.98-1.04)	5315 (39.7)	0.98 (0.94-1.02)	8223 (61.5)	0.97 (0.94-1.00)
Other or unknown	424 (65.4)	0.95 (0.85-1.06)	513 (79.2)	0.99 (0.90-1.09)	182 (28.1)	1.01 (0.85-1.20)	267 (41.2)	0.88 (0.76-1.01)
Intersectionality								
Heterosexual								
Men	6335 (83.3)	1 [Reference]	7105 (93.4)	1 [Reference]	3271 (43.0)	1 [Reference]	5004 (65.8)	1 [Reference]
TQNB	23 (69.7)	0.82 (0.67-1.01)	31 (93.9)	0.99 (0.90-1.08)	15 (45.5)	1.05 (0.86-1.28)	17 (51.5)	0.80 (0.61-1.06)
Women	9391 (84.3)	0.99 (0.98-1.00)	10 548 (94.7)	1.02 (1.01-1.03)	4546 (40.8)	0.98 (0.95-1.01)	6966 (62.6)	0.97 (0.95-0.99)
LGB								
Men	1020 (79.6)	0.90 (0.86-0.94)	1254 (97.9)	1.03 (1.02-1.04)	547 (42.7)	0.90 (0.84-0.96)	805 (62.8)	0.95 (0.90-1.00)
TQNB	60 (50.0)	0.56 (0.47-0.67)	115 (95.8)	1.01 (0.98-1.04)	45 (37.5)	0.66 (0.54-0.80)	39 (32.5)	0.71 (0.58-0.87)
Women	762 (74.9)	0.85 (0.81-0.89)	982 (96.6)	1.02 (1.01-1.03)	416 (40.9)	0.88 (0.82-0.94)	573 (56.3)	0.89 (0.84-0.94)
Other or unknown	1796 (71.5)	0.93 (0.90-0.96)	2131 (84.8)	0.97 (0.95-0.99)	745 (29.7)	0.93 (0.88-0.98)	1305 (51.9)	0.92 (0.89-0.96)

Abbreviations: ARR, adjusted relative ratio; LGB, lesbian, gay, or bisexual; LGBT+, lesbian, gay, bisexual, or transgender; TQNB, transgender, queer, or nonbinary.

^a^ “My institution is welcoming of lesbian, gay, bisexual, and transgender individuals.”

^b^ “I am comfortable working with my lesbian, gay, bisexual, and transgender colleagues.”

^c^ “There is lesbian, gay, bisexual, and transgender visibility at my institution.” (This item was not included on the 2021 survey.)

^d^ “The culture of my institution values respect and tolerance for all.”

^e^ Results are presented as the number (percentage) of individuals who responded “strongly agree” or “agree” to each question.

^f^ Models were adjusted for survey year, generational age group, race and ethnicity, main affiliation, and primary work location. Sexual orientation models were additionally adjusted for gender identity. Gender identity models were additionally adjusted for sexual orientation. All models were weighted by race and ethnicity, job position, and sex to account for nonresponse.

### Perceptions of Institutional Culture

Respondents identifying as LGB were less likely to agree that the institution’s culture valued respect and tolerance for all (ARR, 0.93; 95% CI, 0.90-0.96) compared with heterosexual respondents (Table 3). TQNB respondents were less likely to agree compared with men (ARR, 0.75; 95% CI, 0.58-0.97), and LGB and TQNB respondents were less likely to agree compared with heterosexual men (ARR, 0.71; 95% CI, 0.58-0.87). The greatest difference was generally observed among staff (eTable 6 in Supplement 1). No meaningful differences were found from 2015 to 2023 (eTable 7 in Supplement 1).

### Job Change Considerations

**Figure 2.**
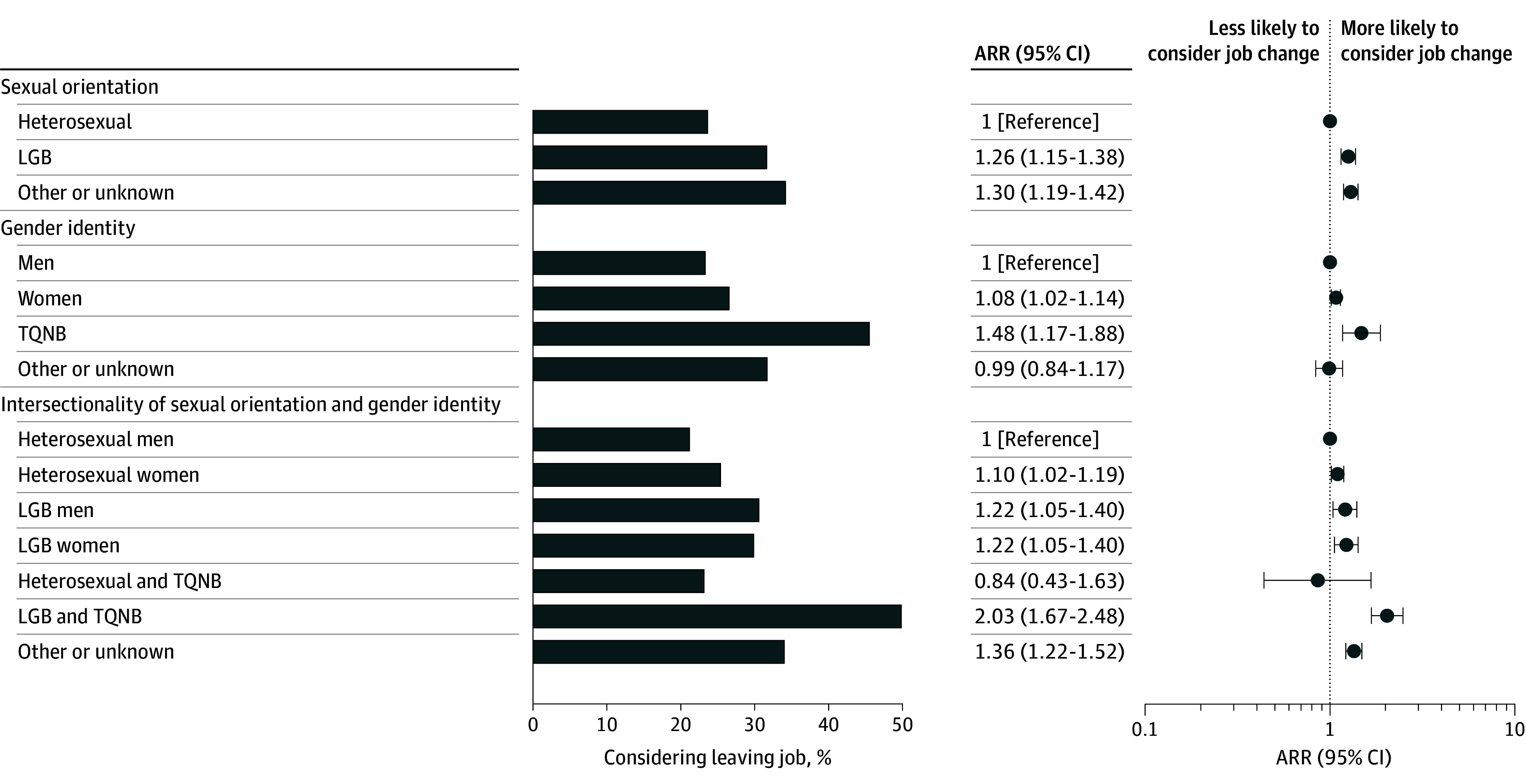
Considerations When Changing Jobs by Sexual Orientation, Gender Identity, and the Intersection of Sexual Orientation and Gender Identity Among Faculty and Staff Models were adjusted for survey year, generational age group, race and ethnicity, main affiliation, and primary work location. Sexual orientation models were additionally adjusted for gender identity. Gender identity models were additionally adjusted for sexual orientation. All models were weighted by race and ethnicity, job position, and sex to account for nonresponse. ARR indicates adjusted relative ratio; LGB, lesbian, gay, or bisexual; TQNB, transgender, queer, or nonbinary.

Respondents identifying as LGB were more likely than respondents identifying as heterosexual to report that they had considered changing jobs due to inappropriate, disruptive, or unprofessional behavior by a coworker or supervisor (LGB, 561 [31.9%]; heterosexual, 3773 [23.9%]; ARR, 1.26; 95% CI, 1.15-1.38) (Figure 2). Similarly, respondents identifying as TQNB were more likely than respondents identifying as men to report that they had considered changing jobs (ARR, 1.48; 95% CI, 1.17-1.88). Respondents identifying as women were also more likely than respondents identifying as men to report that they had considered changing jobs (ARR, 1.08; 95% CI, 1.02-1.14). In addition, compared with respondents identifying as heterosexual men, those identifying as LGB and TQNB were more likely to report that they had considered changing jobs (heterosexual men, 1367 [21.4%]; LGB and TQNB, 52 [50.0%]; ARR, 2.03; 95% CI, 1.16-2.48). The percentage of individuals who reported considering changing jobs increased from 22.3% in 2015 to 27.1% in 2023; however, no meaningful difference was observed from 2015 to 2023 (eTable 8 in Supplement 1).

## Discussion

Our analysis of nearly a decade’s worth of DES data revealed substantial disparities in both engagement within the broader workforce community and perceptions of inclusion among the sexual and gender minority community in a large urban medical school. Specifically, we observed that individuals who identified as LGB and/or TQNB consistently had a lower sense of vision and purpose, camaraderie, and appreciation within the institution compared with respondents who did not identify as being from a sexual or gender minority group. These constructs are central to measuring institutional engagement and were designed to relate to overall productivity and retention.^6^ Additionally, our institution augmented the DES with additional questions specific to the LGBT+ community. Individuals who identified as being from both a sexual and a gender minority group were 44% less likely to feel that the institution was welcoming of LGBT+ individuals and 34% less likely to feel that there was visibility for LGBT+ individuals within the institution. Likewise, 50.0% of individuals who identified as being from both a sexual and a gender minority group reported that they had considered changing jobs, which was more than twice the rate among individuals who identified as heterosexual men.

We also found that LGBT+ individuals were substantially more likely to report considering changing jobs due to inappropriate, disruptive, or unprofessional behavior by a coworker or supervisor compared with their colleagues who did not identify as a sexual or gender minority. Our findings suggest that LGBT+ individuals are particularly at risk of leaving the workplace. While individuals leave a workplace for various reasons, turnover due to the institution’s culture is one preventable cause.^14^ As others have noted, harassment in academic medicine is pervasive, which may be due to the inherent hierarchies built into academic medicine and a culture that supports harassment.^15^ Greater efforts are needed to change the environment and ensure accountability to the ethical standards that promote safety.

Starting with the development of the interprofessional Penn Medicine Program for LGBT Health in 2012, Penn established a clear mission and strategic plan to become a leader in LGBT+ patient care, education, research, and advocacy both locally and nationally (eFigure in Supplement 1).^10^ Consequently, Penn has implemented a wide range of initiatives focused on LGBT+ faculty, students, staff, and patients.^10^ Additionally, in 2021, Penn recognized the need for a greater focus on elevating voices and creating an environment with equitable engagement and implemented a whole-scale planning process.^16^ While we do not have data from before the initiation of the Penn Medicine Program for LGBT Health, our data from this study suggest that consistent and substantial disparities in the perceptions of institutional climate and visibility among LGBT+ community members remain, and thus, additional focused and specific initiatives are still needed to improve the climate. For example, identifiable support groups were initiated to voice the interests of the LGBT+ community. However, additional research is needed to assess specific institutional programs, such as employee resource groups,^17^ and whether they promote positive and sustained changes in the perceptions of institutional culture and visibility for LGBT+ individuals. More support may be needed to amplify the concerns of specific demographic subgroups to effectively address the needs of those within each group. Additionally, in the past, the program was largely outwardly focused on the health and care of LGBT+ patients. We recognize that a priority moving forward needs to be the LGBT+ people within the health care system.

Overall, we did not observe appreciable differences in any of our findings from 2023 vs 2015. There may have been some gains for the items assessing engagement and institutional culture from 2015 to 2018, but whether this was a statistical anomaly or a tangible gain that was not sustained is unclear given the bigger picture of no overall gains. While it is difficult to understand all of the contributing factors, the institutional changes that occurred at Penn also took place alongside many external factors that have affected health equity, including the *Dobbs v Jackson* decision, a wave of antitransgender legislation across the country, the global COVID-19 pandemic, and the murder of George Floyd.^18,19,20^ Not only has the *Dobbs v Jackson* ruling had direct implications for the reproductive health care of LGBT+ people, but it also has had broader implications for established rights of LGBT+ people.^21,22^ Additionally, antitransgender laws negatively affect the health and well-being of transgender people and have implications for inclusion in learning environments.^23,24^ More recent initiatives to restrict diversity, equity, and inclusion initiatives are also likely to negatively impact LGBT+ people at our institution, and it is essential that the initiatives are maintained and strengthened in response.

### Strengths and Limitations

A strength of our study was the large sample size and the ability to leverage 4 surveys spanning 8 years. Penn specifically added questions about LGBT+ institutional inclusivity, which are not universally included in the DES. Another strength was the subanalyses distinguishing among faculty, staff, and students or fellows, in which we observed a greater disparity in responses among staff. This is important to note, as much of the literature surrounding academic medical centers has focused on either physician or student perspectives, with less focus on staff who are essential to the success of health care systems. In addition, we separately assessed gender identity and sexual orientation, which allowed for assessment of each group and joint associations.

This study also has limitations. Our findings may not be generalizable to other settings. Our data were collected from a diverse set of hospitals and health science schools at Penn. These findings should be interpreted within the context of an organization that has actively developed specific initiatives to engage all segments of the community.^10,16^ A second limitation is nonresponse. We estimated an overall response rate of approximately 16%. All analyses were weighted to mitigate potential selection bias. Weights were calculated for each participant to adjust proportions in the observed sample to resemble those of the Perelman School of Medicine. However, because not all participants were from the Perelman School of Medicine and we lacked demographic information for the broader target population, these weights may not fully account for potential bias.

## Conclusions

In this survey study, findings revealed inequalities based on sexual orientation and gender identity across many workplace engagement, inclusivity, and culture domains in an academic medical community. Not only did sexual and gender minority community members not feel as visible or welcomed by the institution, but they were also more likely to consider changing jobs. Given the potential for attrition and the downstream impacts of clinician-patient identity concordance on patient care^25^ as well as the critical role of LGBT+ mentorship in supporting trainees and strengthening workforce representation,^26,27^ this work has implications for both health care delivery and training of the next generation of health care workers.

## Notes

Supplement 1. **eTable 1.** Measures of Institutional Engagement and Their Corresponding Constructs and Questions From the Diversity Engagement Survey **eTable 2.** Sexual Orientation and Gender Identity Response Options by Year and Final Categorizations Used in Analyses **eTable 3.** Demographic Characteristics Overall of Survey Respondents After Weighting to Account for Nonresponse **eTable 4.** Associations of Respondent Sexual Orientation With Measures of Institutional Engagement by Job Position **eTable 5.** Associations of Respondent Sexual Orientation With Measures of Institutional Engagement by Survey Year **eTable 6.** Associations of Respondent Sexual Orientation With Measures of LGBT+ Institutional Inclusivity and Institutional Culture by Job Position **eTable 7.** Associations of Respondent Sexual Orientation With Measures of LGBT+ Institutional Inclusivity and Institutional Culture by Survey Year **eTable 8.** Associations of Respondent Sexual Orientation With Measure of Considering Changing Jobs Among Faculty and Staff by Survey Year **eFigure.** Overview of Events and Activities for the Penn Medicine Program for LGBT+ Health **eMethods.** Additional Information on Weighting to Account for Selection Bias Due to Nonresponse

Supplement 2.
Data Sharing Statement
