# Supplementary material for: Perceptions of Institutional Engagement and Inclusion by Sexual Orientation and Gender Identity
Source: JAMA Netw Open. 2025 Jun 4;8(6):e2513772. doi: 10.1001/jamanetworkopen.2025.13772 (PMC12138678; doi:10.1001/jamanetworkopen.2025.13772)
Supplement: Supplement 1. — eTable 1. Measures of Institutional Engagement and Their Corresponding Constructs and Questions From the Diversity Engagement Survey eTable 2. Sexual Orientation and Gender Identity Response Options by Year and Final Categorizations Used in Analyses eTable 3. Demographic Characteristics Overall of Survey Respondents After Weighting to Account for Nonresponse eTable 4. Associations of Respondent Sexual Orientation With Measures of Institutional Engagement by Job Position eTable 5. Associations of Respondent Sexual Orientation With Measures of Institutional Engagement by Survey Year eTable 6. Associations of Respondent Sexual Orientation With Measures of LGBT+ Institutional Inclusivity and Institutional Culture by Job Position eTable 7. Associations of Respondent Sexual Orientation With Measures of LGBT+ Institutional Inclusivity and Institutional Culture by Survey Year eTable 8. Associations of Respondent Sexual Orientation With Measure of Considering Changing Jobs Among Faculty and Staff by Survey Year eFigure. Overview of Events and Activities for the Penn Medicine Program for LGBT+ Health eMethods. Additional Information on Weighting to Account for Selection Bias Due to Nonresponse [file jamanetwopen-e2513772-s001.pdf]

Supplementary Online Content

Hinkle SN, Okeh CC, Ulloa-Pérez E, et al. Perceptions of institutional engagement and inclusion by sexual orientation and gender identity. *JAMA Netw Open*. 2025;8(6):e2513772. doi:10.1001/jamanetworkopen.2025.13772

eTable 1. Measures of Institutional Engagement and Their Corresponding Constructs and Questions From the Diversity Engagement Survey
eTable 2. Sexual Orientation and Gender Identity Response Options by Year and Final Categorizations Used in Analyses
eTable 3. Demographic Characteristics Overall of Survey Respondents After Weighting to Account for Nonresponse
eTable 4. Associations of Respondent Sexual Orientation With Measures of Institutional Engagement by Job Position
eTable 5. Associations of Respondent Sexual Orientation With Measures of Institutional Engagement by Survey Year
eTable 6. Associations of Respondent Sexual Orientation With Measures of LGBT+ Institutional Inclusivity and Institutional Culture by Job Position
eTable 7. Associations of Respondent Sexual Orientation With Measures of LGBT+ Institutional Inclusivity and Institutional Culture by Survey Year
eTable 8. Associations of Respondent Sexual Orientation With Measure of Considering Changing Jobs Among Faculty and Staff by Survey Year
eFigure. Overview of Events and Activities for the Penn Medicine Program for LGBT+ Health
eMethods. Additional Information on Weighting to Account for Selection Bias Due to Nonresponse

This supplementary material has been provided by the authors to give readers additional information about their work.

© 2025 Hinkle SN et al. *JAMA Network Open*.

eTable 1. Measures of institutional engagement and their corresponding constructs and questions from the Diversity Engagement Survey.

Score	Construct	Question[a]
**Vision/Purpose** Range 10-50	**Common Purpose**	I feel that my work or studies contributes to the mission of the institution
		I feel connected to the vision, mission and values of this institution
	**Access to Opportunity**	This last year, I have had opportunities at work/school to develop professionally
		There is someone at work/school who encourages my development
	**Equitable Reward and Recognition**	I receive recognition and praise for my good work similarly to others who do good work at this institution
		In my institution I am confident that my accomplishments are compensated similarly to others who have achieved their goals
	**Cultural Competence**	In this institution I have opportunities to work successfully in settings with diverse colleagues
		I believe my institution manages diversity effectively
		In my institution, I receive support for working with diverse groups and working in cross-cultural situations
		In this institution, there are opportunities for me to engage in service and community outreach
**Camaraderie** Range 6-30	**Trust**	I trust my institution to be fair to all employees and students
		If I raised a concern about discrimination, I am confident my institution would do what is right
		I believe that in my institution harassment is not tolerated
	**Sense of Belonging**	At work/school, my opinions matter
		I consider at least one of my co-workers or fellow students to be a trusted friend
		I feel that I am an integral part of my department or school
**Appreciation** Range 6-30	**Appreciation of Individual Attributes**	I am valued as an individual by my institution
		Someone at work/school seems to care about me as an individual
		The culture of my institution is accepting of people with different ideas
	**Respect**	The leadership of my institution is committed to treating people respectfully
		In my institution, I experience respect among individuals and groups with various cultural differences
		I believe that my institution reflects a culture of civility

[a] All questions were scored on a five-point Likert scale (5 = strongly agree and 1 = strongly disagree) and summed to create the total score.

© 2025 Hinkle SN et al. *JAMA Network Open*.

eTable 2. Sexual orientation and gender identity response options by year and final categorizations utilized in analyses.

	2015	2018	2021	2023
**Sexual Orientation**	Heterosexual	Heterosexual	Heterosexual	Heterosexual
	Gay	Gay	Gay	Gay
	Lesbian	Lesbian	Lesbian	Lesbian
	Bisexual	Bisexual	Bisexual	Bisexual
	Questioning			
	Other (please specify)	Different Orientation (please specify)	Different Orientation (please specify)	Different Orientation (please specify)
**Gender Identity**	Male[a]	Male[a]	Male[a]	Man
	Female[a]	Female[a]	Female[a]	Woman
	Transgender	Trans Male/Trans Man	Trans Male/Trans Man	Trans Man
		Trans Female/Trans Woman	Trans Female/Trans Woman	Trans Woman
		Gender Queer/Gender Non-Conforming	Gender Queer/Gender Non-Conforming	Gender Queer/Gender Non-Conforming
	Do not identify as male or female (gender non-conforming, gender queer)	Different Identity (please specify)	Different Identity (please specify)	Different Identity (please specify)

[a] While in 2015, 2018, and 2021, gender identity was collected using inappropriate terms of male and female, we have used men and women throughout the paper corresponding to the appropriate terms used in 2023.

© 2025 Hinkle SN et al. *JAMA Network Open*.

eTable 3. Demographic characteristics overall of survey respondents after weighting[a] to account for non-response.

Characteristic		% (95% CI)
**Sexual Orientation**		
Heterosexual		79.9 (79.4-80.4)
Lesbian, Gay, Bisexual		10.3 (9.9-10.7)
Other/Unknown		9.7 (9.3-10.1)
**Gender Identity**		
Men		40.1 (39.5-40.7)
Women		56.4 (55.8-57.0)
Transgender/Queer/Non-binary		0.7 (0.6-0.8)
Unknown		2.7 (2.5-2.9)
**Intersectionality**		
**Gender Identity**	**Sexual Orientation**	
Men	Heterosexual	32.1 (31.5-32.7)
Women	Heterosexual	47.0 (46.4-47.6)
Men	Lesbian, Gay, Bisexual	5.4 (5.1-5.7)
Women	Lesbian, Gay, Bisexual	4.3 (4.0-4.6)
Trans/Queer/Non-binary	Heterosexual	0.1 (0.1-0.1)
Trans/Queer/Non-binary	Lesbian, Gay, Bisexual	0.5 (0.4-0.6)
Other/Unknown		10.6 (10.2-11)
**Age Group**		
1922-1944		0.8 (0.7-0.9)
1945-1964		22.6 (22.1-23.1)
1965-1980		33.7 (33.1-34.3)
1981-		40.1 (39.5-40.7)
Unknown		2.9 (2.7-3.1)
**Race/Ethnicity**		
Asian		16.5 (16.0-17.0)
Hispanic/Latino		3.6 (3.4-3.8)
Non-Hispanic Black		12.0 (11.6-12.4)
Non-Hispanic White		57.9 (57.3-58.5)
Multiple races/ethnicities		3.9 (3.7-4.1)
Other races/ethnicities/Unknown[b]		6.1 (5.7-6.3)
**Main Affiliation**		
Staff		39.3 (38.7-39.9)
Standing or Associated Faculty[c]		27.4 (26.8-28.0)
Students or Fellows[d]		17.5 (17.0-18.0)
Other/Unknown		15.8 (15.3-16.3)
**Location of Main Affiliation**		
Pennsylvania Hospital		6.9 (6.6-7.2)
Perelman School of Medicine		14.6 (14.2-15.0)
Philadelphia VA Medical Center		0.4 (0.3-0.5)
School of Nursing		0.4 (0.3-0.5)
University of Pennsylvania Affiliated Hospitals		54.1 (53.5-54.7)
Other/Unknown		23.5 (23.0-24.0)

[a] Weights based on the characteristics the Perelman School of Medicine from the Penn Community Survey for years 2015, 2018, 2021, and 2023.
[b] Other category includes: 'Native Hawaiian/Other Pacific Islander', 'American Indian/Alaska Native', as well as people who wrote in other responses.
[c] Includes leadership
[d] Includes undergraduate students, graduate students, professional students, interns, residents, clinical fellows, postdoctoral fellows.

© 2025 Hinkle SN et al. *JAMA Network Open*.

eTable 4. Associations of respondent sexual orientation with measures of institutional engagement by job position.

Sexual Orientation	Staff	Graduate Students and Postdocs	Standing or Associated Faculty	Other/Missing
**Vision and Purpose**				
(1) Heterosexual[a]	39.1 (7.6)	39.8 (7.8)	39.9 (7.3)	39.6 (7.7)
(2) Lesbian, Gay, Bisexual[a]	37.5 (8.1)	38.9 (7.4)	39.2 (7.6)	38.9 (7.8)
(3) Other/Unknown[a]	37.1 (8.6)	37.8 (8.7)	36.8 (9.2)	37.3 (8.4)
Adjusted Difference 2 *vs.* 1[b]	-1.8 (-2.3, -1.3)	-1.0 (-2.2, 0.3)	-0.6 (-1.9, 0.7)	-0.7 (-2.3, 0.8)
Adjusted Difference 3 *vs.* 1[b]	-0.7 (-1.2, -0.1)	-0.2 (-1.7, 1.3)	-0.7 (-2.1, 0.7)	-0.3 (-1.6, 1.1)
**Camaraderie**				
(1) Heterosexual[a]	23.4 (4.8)	23.2 (5.0)	23.9 (4.6)	23.9 (4.8)
(2) Lesbian, Gay, Bisexual[a]	21.9 (5.1)	21.9 (5.1)	23.2 (4.9)	23.1 (5.1)
(3) Other/Unknown[a]	22.1 (5.5)	22.4 (5.4)	21.5 (5.9)	22.0 (5.4)
Adjusted Difference 2 *vs.* 1[b]	-1.3 (-1.6, -1.0)	-1.1 (-1.9, -0.4)	-0.6 (-1.4, 0.2)	-0.8 (-1.7, 0.2)
Adjusted Difference 3 *vs.* 1[b]	-0.4 (-0.8, -0.1)	0.4 (-0.6, 1.3)	-0.8 (-1.7, 0.0)	-0.3 (-1.2, 0.5)
**Appreciation**				
(1) Heterosexual[a]	23.8 (4.8)	24.0 (4.9)	24.3 (4.6)	24.3 (4.8)
(2) Lesbian, Gay, Bisexual[a]	22.8 (5.1)	23.0 (4.5)	23.7 (4.9)	23.5 (5.3)
(3) Other/Unknown[a]	22.5 (5.5)	22.7 (5.7)	21.7 (6.0)	22.5 (5.4)
Adjusted Difference 2 *vs.* 1[b]	-1.1 (-1.4, -0.8)	-0.8 (-1.5, 0.0)	-0.5 (-1.3, 0.3)	-0.8 (-1.7, 0.1)
Adjusted Difference 3 *vs.* 1[b]	-1.3 (-1.7, -1.0)	-1.0 (-1.9, -0.1)	-2.5 (-3.3, -1.7)	-1.8 (-2.6, -1.0)

[a] Results are presented as mean (SE) for each score.
[b] Models were adjusted for survey year, generational age group, race/ethnicity, main affiliation, primary work location, and gender identity. All models were weighted by race, job position, and sex to account for non-response. Results are presented as the adjusted difference in the scores between the respective groups with 95% confidence intervals.

© 2025 Hinkle SN et al. *JAMA Network Open*.

eTable 5. Associations of respondent sexual orientation with measures of institutional engagement by survey year.

Sexual Orientation	2015	2018	2021	2023[c]
**Vision and Purpose**				
(1) Heterosexual[a]	39.7 (6.8)	39.4 (8.0)	39.5 (7.5)	39 (7.6)
(2) Lesbian, Gay, Bisexual[a]	37.8 (7.1)	39.3 (7.4)	37.7 (8.0)	37.9 (8.2)
(3) Other/Unknown[a]	36.3 (8.0)	36.3 (9.6)	37.5 (8.5)	37 (8.4)
Adjusted Difference 2 *vs.* 1[b]	-2.2 (-3.2, -1.2)	0.2 (-2.1, 2.4)	-1.8 (-3.9, 0.3)	-1.1 (-3.3, 1.2)
Adjusted Difference 3 *vs.* 1[b]	-1.0 (-2.7, 0.7)	-1.4 (-5.1, 2.3)	-0.4 (-3.9, 3.1)	0.12 (-3.4, 3.7)
**Camaraderie**				
(1) Heterosexual[a]	23.9 (4.2)	23.6 (5.0)	23.7 (4.7)	23.1 (5.0)
(2) Lesbian, Gay, Bisexual[a]	22 (4.7)	23.1 (4.8)	22.3 (5.1)	21.6 (5.5)
(3) Other/Unknown[a]	21.5 (5.2)	21.6 (5.9)	22.4 (5.4)	21.4 (5.6)
Adjusted Difference 2 *vs.* 1[b]	-1.8 (-2.5, -1.2)	0.02 (-1.4, 1.4)	-1.33 (-2.7, 0.0)	-1.3 (-2.7, 0.2)
Adjusted Difference 3 *vs.* 1[b]	-0.7 (-1.8, 0.43)	-0.92 (-3.3, 1.4)	-0.2 (-2.4, 2.0)	-0.2 (-2.5, 2.0)
**Appreciation**				
(1) Heterosexual[a]	24.1 (4.3)	24 (5.0)	24.2 (4.6)	23.7 (5.0)
(2) Lesbian, Gay, Bisexual[a]	22.7 (4.6)	23.8 (4.6)	22.9 (5.0)	22.7 (5.1)
(3) Other/Unknown[a]	22.2 (4.96)	22 (6.1)	22.8 (5.5)	21.9 (5.5)
Adjusted Difference 2 *vs.* 1[b]	-1.5 (-2.1, -0.9)	0.1 (-1.2, 1.5)	-1.2 (-2.5, 0.1)	-0.9 (-2.3, 0.4)
Adjusted Difference 3 *vs.* 1[b]	-0.5 (-1.6, 0.6)	-0.9 (-3.2, 1.4)	-0.4 (-2.6, 1.8)	-0.3 (-2.6, 1.9)

[a] Results are presented as mean (SE) for each score.
[b] Models were adjusted for survey year, generational age group, race/ethnicity, main affiliation, primary work location, and gender identity. All models were weighted by race, job position, and sex to account for non-response. Results are presented as the adjusted difference in the scores between the respective groups with 95% confidence intervals.
[c] There was no significant difference ($P > 0.05$) in the adjusted relative risk (2 *vs*. 1 or 3 *vs*. 1) between 2015 and 2023 for any of the measures.

© 2025 Hinkle SN et al. *JAMA Network Open*.

eTable 6. Associations of respondent sexual orientation with measures of LGBT+ institutional inclusivity and institutional culture by job position.

Sexual Orientation	Staff	Graduate Students and Postdocs	Standing or Associated Faculty	Other/Missing
**Welcoming**				
(1) Heterosexual[a]	6106 (83.6)	2588 (82.0)	4825 (85.5)	2379 (83.5)
(2) Lesbian, Gay, Bisexual[a]	789 (73.1)	536 (77.8)	358 (81.2)	177 (73.4)
(3) Other/Unknown[a]	680 (73.1)	218 (71.2)	308 (73.3)	423 (64.8)
Adjusted Relative Ratio 2 *vs.* 1[b]	0.85 (0.79, 0.91)	0.89 (0.75, 1.06)	0.92 (0.76, 1.11)	0.87 (0.70, 1.07)
Adjusted Relative Ratio 3 *vs.* 1[b]	0.93 (0.86, 1.01)	0.91 (0.73, 1.13)	0.96 (0.79, 1.17)	0.93 (0.77, 1.13)
**Comfort**				
(1) Heterosexual[a]	6775 (92.7)	2986 (94.6)	5449 (96.6)	2638 (92.6)
(2) Lesbian, Gay, Bisexual[a]	1044 (96.8)	674 (97.7)	433 (98.4)	228 (94.6)
(3) Other/Unknown[a]	793 (85.3)	237 (77.5)	385 (91.7)	525 (80.4)
Adjusted Relative Ratio 2 *vs.* 1[b]	1.02 (0.95, 1.09)	1.02 (0.87, 1.20)	1.01 (0.86, 1.19)	1.01 (0.84, 1.22)
Adjusted Relative Ratio 3 *vs.* 1[b]	0.94 (0.87, 1.01)	0.93 (0.76, 1.14)	1.00 (0.84, 1.20)	0.98 (0.82, 1.16)
**Visibility**				
(1) Heterosexual[a]	3004 (41.1)	1538 (48.8)	2467 (43.7)	886 (31.1)
(2) Lesbian, Gay, Bisexual[a]	393 (36.5)	353 (51.2)	200 (45.5)	71 (29.5)
(3) Other/Unknown[a]	292 (31.4)	102 (33.2)	152 (36.2)	126 (19.3)
Adjusted Relative Ratio 2 *vs.* 1[b]	0.85 (0.76, 0.95)	0.92 (0.72, 1.17)	0.94 (0.73, 1.22)	0.89 (0.64, 1.23)
Adjusted Relative Ratio 3 *vs.* 1[b]	0.90 (0.79, 1.02)	0.87 (0.63, 1.20)	0.98 (0.73, 1.31)	1.01 (0.74, 1.38)
**Culture**				
(1) Heterosexual[a]	4626 (63.3)	1995 (63.3)	3625 (64.3)	1845 (64.8)
(2) Lesbian, Gay, Bisexual[a]	598 (55.4)	426 (61.7)	261 (59.3)	146 (60.6)
(3) Other/Unknown[a]	485 (52.2)	150 (48.9)	230 (54.8)	322 (49.3)
Adjusted Relative Ratio 2 *vs.* 1[b]	0.91 (0.84, 0.99)	0.94 (0.77, 1.15)	0.96 (0.78, 1.19)	0.95 (0.75, 1.21)
Adjusted Relative Ratio 3 *vs.* 1[b]	0.94 (0.85, 1.03)	0.99 (0.77, 1.27)	0.96 (0.76, 1.21)	0.93 (0.75, 1.16)

[a] Results are presented as mean (SE) for each score.
[b] Models were adjusted for survey year, generational age group, race/ethnicity, main affiliation, primary work location, and gender identity. All models were weighted by race, job position, and sex to account for non-response. Results are presented as the adjusted difference in the scores between the respective groups with 95% confidence intervals.

© 2025 Hinkle SN et al. *JAMA Network Open*.

eTable 7. Associations of respondent sexual orientation with measures of LGBT+ institutional inclusivity and institutional culture by survey year.

Sexual Orientation	2015	2018	2021	2023[e]
**Welcoming**				
(1) Heterosexual[a]	2083 (79.2)	3266 (81.6)	6929 (84.4)	3620 (88.1)
(2) Lesbian, Gay, Bisexual[a]	211 (69.6)	461 (77.9)	752 (76.8)	436 (75.8)
(3) Other/Unknown[a]	69 (63.9)	280 (65.4)	867 (70.4)	413 (76.1)
Adjusted Relative Ratio 2 *vs*. 1[b]	0.81 (0.75, 0.88)	0.91 (0.76, 1.08)	0.88 (0.74, 1.04)	0.86 (0.72, 1.03)
Adjusted Relative Ratio 3 *vs*. 1[b]	0.93 (0.82, 1.06)	0.90 (0.69, 1.17)	0.94 (0.73, 1.22)	0.96 (0.74, 1.25)
**Comfort**				
(1) Heterosexual[a]	2463 (93.6)	3712 (92.8)	7762 (94.6)	3910 (95.2)
(2) Lesbian, Gay, Bisexual[a]	300 (98.7)	572 (96.6)	953 (97.3)	554 (96.4)
(3) Other/Unknown[a]	87 (80.6)	356 (83.0)	1047 (85.0)	450 (82.9)
Adjusted Relative Ratio 2 *vs*. 1[b]	1.02 (1.01, 1.03)	1.03 (1.00, 1.06)	1.02 (0.99, 1.05)	1.00 (0.96, 1.04)
Adjusted Relative Ratio 3 *vs*. 1[b]	0.92 (0.84, 1.01)	0.96 (0.79, 1.16)	0.97 (0.80, 1.16)	0.94 (0.78, 1.14)
**Visibility**				
(1) Heterosexual[a]	1775 (67.5)	2829 (70.7)	--[c]	3292 (80.1)
(2) Lesbian, Gay, Bisexual[a]	190 (62.7)	415 (70.1)	--[c]	413 (71.8)
(3) Other/Unknown[a]	64 (59.8)	248 (57.9)	--[c]	359 (66.1)
Adjusted Relative Ratio 2 *vs*. 1[b]	0.85 (0.77, 0.94)	0.93 (0.75, 1.15)	--[c]	0.88 (0.71, 1.08)
Adjusted Relative Ratio 3 *vs*. 1[b]	0.95 (0.81, 1.11)	0.94 (0.68, 1.30)	--[c]	0.92 (0.67, 1.26)
**Culture**				
(1) Heterosexual[a]	2176 (82.7)	3167 (79.1)	6749 (82.2)	--[d]
(2) Lesbian, Gay, Bisexual[a]	227 (74.9)	477 (80.7)	727 (74.2)	--[d]
(3) Other/Unknown[a]	70 (64.8)	272 (63.6)	845 (68.6)	--[d]
Adjusted Relative Ratio 2 *vs*. 1[b]	0.91 (0.84, 0.98)	1.01 (0.86, 1.19)	0.89 (0.76, 1.04)	--[d]
Adjusted Relative Ratio 3 *vs*. 1[b]	0.95 (0.82, 1.11)	0.92 (0.68, 1.25)	0.96 (0.71, 1.30)	--[d]

[a] Results are presented as mean (SE) for each score.
[b] Results are presented as the adjusted difference in the scores between the respective groups with 95% confidence intervals and adjusted for survey year, generational age group, race/ethnicity, main affiliation, primary work location, and gender identity. All models were weighted by race, job position, and sex to account for survey non-response.
[c] Visibility question was not asked in 2021.
[d] Culture question was not asked in 2023.
[e] There was no significant difference (*P* > 0.05) in the adjusted relative risk (2 *vs*. 1 or 3 *vs*. 1) between 2015 and 2023 (2021 for Culture) for any of the measures.

© 2025 Hinkle SN et al. *JAMA Network Open*.

eTable 8. Associations of respondent sexual orientation with measure of considering changing jobs among faculty and staff by survey year.

Sexual Orientation	2015	2018	2021	2023[c]
(1) Heterosexual[a]	416 (21.0)	809 (24.4)	1691 (23.9)	858 (25.1)
(2) Lesbian, Gay, Bisexual[a]	57 (27.7)	97 (28.0)	262 (33.9)	145 (33.4)
(3) Other/Unknown[a]	36 (36.7)	135 (36.8)	358 (32.8)	161 (36.0)
Adjusted Relative Ratio 2 *vs*. 1[b]	1.24 (0.94, 1.63)	1.09 (0.61, 1.93)	1.35 (0.77, 2.36)	1.24 (0.70, 2.18)
Adjusted Relative Ratio 3 *vs.* 1[b]	1.61 (1.20, 2.16)	1.42 (0.77, 2.61)	1.25 (0.69, 2.25)	1.29 (0.71, 2.34)

[a] Results are presented as mean (SE) for each score.
[b] Models were adjusted for survey year, generational age group, race/ethnicity, main affiliation, primary work location, and gender identity. All models were weighted by race, job position, and sex to account for non-response. Results are presented as the adjusted difference in the scores between the respective groups with 95% confidence intervals.
[c] There was no significant difference in the adjusted relative risk (2 *vs.* 1 or 3 *vs*. 1) between 2015 and 2023.

© 2025 Hinkle SN et al. *JAMA Network Open*.

eFigure. Overview of events and activities for the Penn Medicine Program for LGBT+ Health.

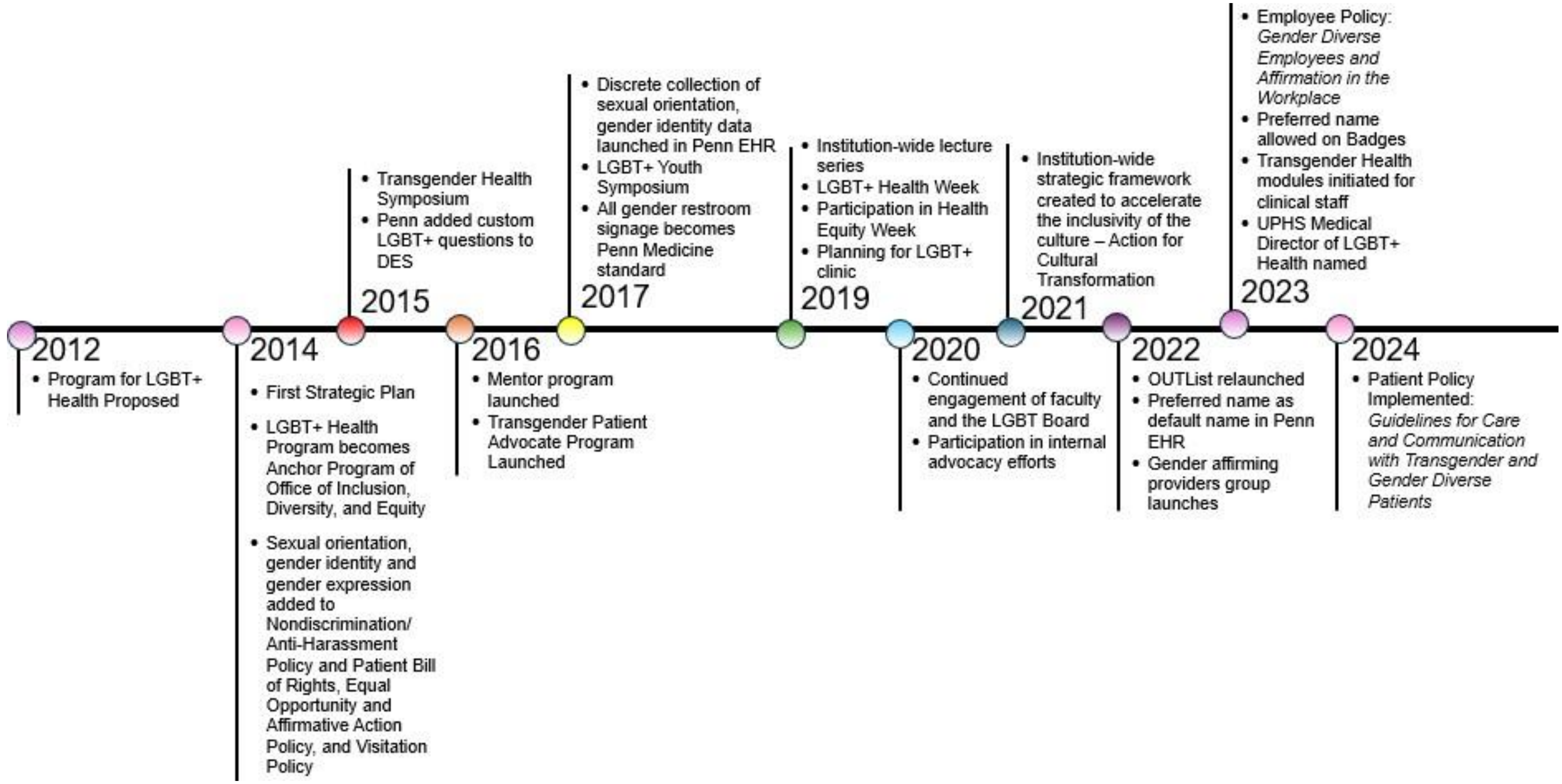

Abbreviations: EHR, electronic health record; LGBT+, Lesbian, Gay, Bisexual, Transgender; UPHS, University of Pennsylvania Health System.

© 2025 Hinkle SN et al. *JAMA Network Open*.

eMethods. Additional information on weighting to account for selection bias due to non-response

The estimated total population for the survey in 2015, 2018, 2021, and 2023 was 18,550, 40,983, 44,000, and 46,000, respectively, with an estimated response rate of 16%, 12%, 24%, and 11% in each of the respective years. To account for potential selection bias due to non-response, we reweighted the survey responses so that the overall frequencies of main affiliation, gender, and race were as reflective as possible of the overall Perelman School of Medicine population in each survey year. We used yearly frequencies for the Perelman School of Medicine from the Penn Community Survey (University of Pennsylvania Diversity Dashboard; https://ira.upenn.edu/penn-numbers/diversity-dashboard) to compute weights for each of the DES survey respondents. The Dashboard publishes information on the distributions of sex and race by job position for each survey year. The reported categories on the dashboard for race and sex are consistent with state and federal guidelines. We used the *anesrake* package in R (Pasek, 2018) to derive the weights.

The Penn Community Survey reported sex as female and male. We used the sex distributions from the Penn Community Survey to reweight the distributions for men and women gender in our Diversity Engagement Survey; we did not reweight individuals within the transgender/queer/non-binary or other/unknown groups and assigned them of a weight of 1. In the Penn Community Survey, overall, 46.1% were reported as men and 53.9% as women. In our Diversity Engagement Survey sample the percentage of men before versus after weighing 25.2% *vs*. 40.1%, while the percentage of women was 71.3% *vs*. 56.4%. The percentage of Transgender/Queer/Non-binary was unchanged at 0.7% before and after weighting.

The Penn Community Survey reported race as Asian, Black, Hispanic, and Indigenous, and White; however, within the student and postdoc positions the race category also included "International". In the Penn Community Survey, the percentages of Asian, Hispanic/Black/Indigenous, White, and International were 18.9%, 13.1%, 62.6%, and 5.4%. The Diversity Engagement Survey does not contain that option for race. To solve this lack of overlap, for students and postdocs we distributed the frequencies from the "International" category to the remaining categories of White, Asian, and Black or Hispanic assuming that the race proportions would be equal to those observed in the Standing or Associated Faculty category (which do not contain international category). In our Diversity Engagement Survey sample, the race and ethnicity percentages before versus after weighting were: Asian 7.8% *vs*. 16.5%; Hispanic/Latino 3.9% *vs*. 3.6%; Non-Hispanic Black 15.5% *vs*. 12.0%; Non-Hispanic White 62.9% *vs*. 57.9%; multiple races/ethnicities 3.6% *vs*. 3.9%; other races/ethnicities/unknown 6.3% *vs*. 6.1%.

© 2025 Hinkle SN et al. *JAMA Network Open*.
