# Supplementary material for: Perceptions of Institutional Engagement and Inclusion by Sexual Orientation and Gender Identity
Source: JAMA Netw Open. 2025 Jun 4;8(6):e2513772. doi: 10.1001/jamanetworkopen.2025.13772 (PMC12138678; doi:10.1001/jamanetworkopen.2025.13772)
Supplement: Supplement 2. — Data Sharing Statement [file jamanetwopen-e2513772-s002.pdf]

Data Sharing Statement

Hinkle. Perceptions of Institutional Engagement and Inclusion by Sexual Orientation and Gender Identity. *JAMA Netw Open*. Published June 04, 2025.
doi:10.1001/jamanetworkopen.2025.13772

Data
Data available: No

Additional Information
Explanation for why data not available: The authors of this paper are not able to share the data per their data use agreement with the University of Massachusetts Diversity Engagement Survey. Researchers interested in using the data should contact the University of Massachusetts Diversity Engagement Survey (https://www.diversityengagementsurvey.com/)
